# scRNASequest: an ecosystem of scRNA-seq analysis, visualization, and publishing

**DOI:** 10.1186/s12864-023-09332-2

**Published:** 2023-05-02

**Authors:** Kejie Li, Yu H. Sun, Zhengyu Ouyang, Soumya Negi, Zhen Gao, Jing Zhu, Wanli Wang, Yirui Chen, Sarbottam Piya, Wenxing Hu, Maria I. Zavodszky, Hima Yalamanchili, Shaolong Cao, Andrew Gehrke, Mark Sheehan, Dann Huh, Fergal Casey, Xinmin Zhang, Baohong Zhang

**Affiliations:** 1 Research Data Sciences, Translational Biology, Biogen Inc., Cambridge, MA 02142 USA; 2 Data Science, BioInfoRx Inc., Madison, WI 53719 USA

**Keywords:** Single-cell RNA-seq, Single-nucleus RNA-seq, Transcriptome, Data integration, Cell-type label transfer, Batch correction

## Abstract

**Background:**

Single-cell RNA sequencing is a state-of-the-art technology to understand gene expression in complex tissues. With the growing amount of data being generated, the standardization and automation of data analysis are critical to generating hypotheses and discovering biological insights.

**Results:**

Here, we present scRNASequest, a semi-automated single-cell RNA-seq (scRNA-seq) data analysis workflow which allows (1) preprocessing from raw UMI count data, (2) harmonization by one or multiple methods, (3) reference-dataset-based cell type label transfer and embedding projection, (4) multi-sample, multi-condition single-cell level differential gene expression analysis, and (5) seamless integration with cellxgene VIP for visualization and with CellDepot for data hosting and sharing by generating compatible h5ad files.

**Conclusions:**

We developed scRNASequest, an end-to-end pipeline for single-cell RNA-seq data analysis, visualization, and publishing. The source code under MIT open-source license is provided at https://github.com/interactivereport/scRNASequest. We also prepared a bookdown tutorial for the installation and detailed usage of the pipeline: https://interactivereport.github.io/scRNAsequest/tutorial/docs/. Users have the option to run it on a local computer with a Linux/Unix system including MacOS, or interact with SGE/Slurm schedulers on high-performance computing (HPC) clusters.

**Supplementary Information:**

The online version contains supplementary material available at 10.1186/s12864-023-09332-2.

## Background

With the development of next-generation sequencing (NGS) technologies, researchers have started to focus more on the characterization at the single cell level [1, 2]. The single-cell RNA sequencing (scRNA-seq) technique, with the generation of expression profiles on individual cells, preserves both the representative biological patterns and heterogeneity, especially for some rare but informative subtypes [3–5]. As such, it has provided many novel insights into diversified complex biological systems, such as the brain [6, 7], lung [8], immune system [9–11], reproductive system [12–14], and multiple cancer-related tissues [15–20].

A general workflow for scRNA-seq analysis, with the goal of data exploration and interpretation, starts with read alignment and quality control (QC). These can give a quantitative estimation of genome mapping rate, unique molecular identifier (UMI) counts, mitochondria read percentage, and overall data quality. Following that, data composed of multiple samples and conditions requires batch effect correction and normalization [3, 21, 22]. Next, to achieve a basic understanding of the main trends and patterns in the dataset, some processing steps, including dimensionality reduction, feature selection, and clustering, are implemented [21, 23–26]. Furthermore, the diversified downstream analysis allows the researchers to uncover gene expression differences between different groups, investigate changes in gene regulatory networks, and infer trajectories of distinct cell lineages [3, 27–29].

To make the best use of scRNA-seq data, multiple analysis tools have been developed to optimize the individual steps [30]. For quality control, there are Cell Ranger (10 × Genomics) and Kallisto/bustools [31]. For preprocessing and harmonization, there are Seurat [32, 33], Scanpy [34], Harmony [35], and LIGER [36, 37]. The term, harmonization, was used for scRNA-seq data to emphasize that the data come from different sources [38]**.** Harmonization also differs from batch correction because it usually corrects the data on a 2-dimensional UMAP or t-SNE space rather than adjusting the UMI counts directly. Furthermore, Seurat and Scanpy can also be used to perform further data processing and downstream analysis [39]. However, having to choose different methods for the different steps, users are faced with the daunting tasks of tool comparisons, individual parameter optimizations, and the integration of multiple packages while assuring the reproducibility of the results. Thus, an automatic workflow with the combination of state-of-the-art methods, equipped with metrics evaluations and visualizations to help make decisions, will significantly benefit scRNA-seq data analyses by making them simpler and faster.

A similar R-based workflow, scFlow, has been developed to standardize the whole framework, which allows QC, integration, clustering, cell type annotation, differential expression (DE) analysis, and pathway analysis, but it does not provide downstream data visualization and sharing options [40]. Additionally, when the number of cells is over one million, R has a limitation in reading in such a large (one million by 20 k gene) matrix and creating a sparse matrix. To overcome these limitations, we developed scRNASequest, an end-to-end pipeline for single-cell RNA-seq analysis. Our implementation using both Python and R allows users to handle more than one million cells [34, 41]. Moreover, the pipeline is also customizable for parameter adjustment and diverse output formats, including a bookdown report [42], a slide deck presentation, and an integrated h5ad file, which can be visualized in cellxgene VIP [43, 44] and integrated into the CellDepot data repository [45]. Detailed comparisons of scRNASequest with similar tools have been summarized, highlighting unique advantages of our pipeline (Table S1). Overall, scRNASequest simplifies and generalizes the single-cell RNA-seq workflow, which offers the opportunity to analyze large datasets in a standard and time-efficient manner.

## Implementation

**Fig. 1:**
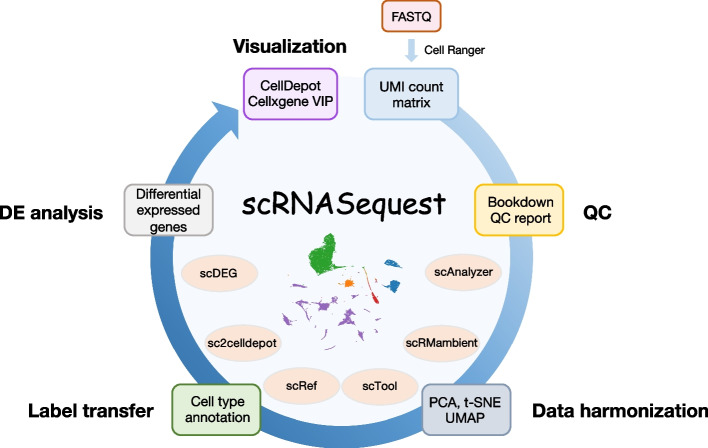
Overview of the scRNASequest workflow

The scRNAsequest is a generalized pipeline including the critical steps necessary to perform an end-to-end scRNA-seq analysis (Fig. 1, Table 1). It is fully compatible with SGE and Slurm high-performance computing (HPC) clusters, and users can specify the HPC type and CPU number in the configuration file (config.yml). We offer flexible installation options using Conda or Docker, with the latter ensuring better distribution of the pipeline across different operating systems, including Linux and MacOS. Moreover, detailed instruction has been included in both the GitHub webpage and online tutorial, coupled with a demonstration dataset. Before initiating scRNAsequest, Cell Ranger has to be run on the raw sequencing data to generate basic quality metrics and the UMI count matrix [46]. Since our pipeline does not use gene annotation or any species-specific information, it requires the users to choose the correct species when running Cell Ranger.

**Table 1:** Pipeline scripts and their function

Command	Description	Input	Output
scAnalyzer	Main program to perform full scRNA-seq data analysis with QC and data harmonization	A path to analysis config file (config.yml)	Final analysis results in h5ad and h5Seurat format
scDEG	Program to perform DEG analysis between two phenotypes within each cluster of an annotation (such as cell types)	A path to a DEG config file (config_DEG.yml)	One DEG table for each cluster and an SQLite db file of all comparisons
scRef	Program to create Seurat 'Azimuth' references	A path to a reference config file (config_ref.yml)	An RDS object with 'Azimuth' reference for scAnalyzer
sc2celldepot	Program to transfer analyzed data into h5ad for the cellxgene VIP (CellDepot) loading	A path to a data config file (config_convert.yml)	An h5ad file
scTool	Tool to add, remove or export express/annotation from an h5ad file	A path to an h5ad file	A modified h5ad file or a csv file
scRMambient	Program to remove ambient RNA by CellBender	A path to a sample metadata file containing paths to raw (unfiltered) UMI along with a few CellBender parameters	CellBender filtered UMI counts in h5 format

### Ambient RNA removal

Ambient RNAs are contaminating RNAs in the cell suspension during scRNA-seq sample preparation [47]. Single-nucleus RNA-seq, with cell lysed during the nuclei extraction step, is particularly prone to ambient RNA contamination. In a recent paper [48], CellBender [49] outperforms other tools including DecontX [50] and SoupX [51]. Thus, before running the full workflow, we incorporated an optional step called scRMambient to remove ambient RNAs using CellBender.

### Quality control and filtering of single-cell RNA-seq data

The pipeline starts with setting several parameters used for assessing data quality control defined in the configuration file (config.yml, Table 2). Multiple plots are generated to visually aid this QC assessment, indicating the number of genes detected, total UMI counts, and percentage of mitochondrial reads at a single-cell level. If the Cell Ranger-generated summary of sequencing metrics is available (metrics_summary.csv), the pipeline will also generate figures based on that. This step was implemented using Scanpy [34] and employs multiple adjustable parameters to filter out low-quality data (Table 3). Users can remove low-expression genes detected only in a small number of cells (min.cells = 3), low-quality cells with a limited number of genes expressed (min.features = 50), or potential doublets with too many counts (highCount.cutoff = 10,000) and an unusually high number of genes detected (highGene.cutoff = 3,000). The numbers in parentheses are the empirically established default values of some of the adjustable parameters used at this step.

**Table 2:** Configuration files and their usage

File name	File description and usage	Parameters
config.yml	Main configuration file required for running the pipeline. This file can be generated by running scAnalyzer with a directory provided.	1, Project-related information: project name, project title 2, QC: Percentage cutoff of mitochondria reads and user-defined gene groups, cell and gene filtering criteria 3, Run setting: SGE or Slurm, number of CPUs, harmonization methods, scaling factor 4, Path to sample metadata information, reference file for label transfer in Azimuth format [32], output directory, path to the DE comparison file 5, Settings for DE analysis
sys.yml	System configuration file under the pipeline source (src) directory. This file only needs to be set up once after pipeline installation.	1, Path to a directory to store h5ad files for cellxgene VIP loading 2, Path to all references for cell type label transfer 3, Detailed information about the reference datasets 4, Miscellaneous settings of the pipeline
config_DEG.yml	Configuration file for running the DE analysis separately. This file can be generated by running scDEG with a directory provided. Several items are the same as the config.yml file.	1, Path to a UMI count matrix in h5ad or RDS format 2, Path to a sample metadata information file 3, Run setting: SGE or Slurm 4, Path to the DE comparison file 5, Settings for DE analysis
config_ref.yml	Configuration file for building the reference for cell type label transfer. This file can be generated by running scRef with a directory provided.	1, Path to an h5ad file containing UMI count matrix and cell annotation 2, Detailed information about this reference, including which labels to be used for future label transfer
config_convert.yml	Configuration file for converting public data to the standardized format for cellxgene VIP and CellDepot loading. This file can be generated by running scRef with a directory provided.	1, Path to the data file in h5ad or RDS format 2, Miscellaneous setting on the outputs

**Table 3:** Summary of cell and gene counts before and after each filtering step

Parameter	Cutoff	Cell count	Gene number
MT	20%	40,532	31,040
min.cell	3	40,532	25,115
min.features	50	40,532	25,115
highGene.cutoff	3,000	29,561	25,115
highCount.cutoff	10,000	29,511	25,115

### Data harmonization and result evaluation

The scRNASequest pipeline takes filtered UMI count matrices from different samples or batches to create an integrated data object through predefined state-of-the-art methods [52, 53], including Seurat [32], Seurat RPCA [33], Harmony [35], and LIGER [36]. Log normalization or SCtransform [54] can be applied to the UMI matrix of each sample to obtain the normalized expression levels per cell and per gene. In addition, we implemented the kBET [55] (Fig. S1A) and silhouette [55–57] (Fig. S1B) metrics to evaluate the performance of different harmonization methods against samples or batches.

### Reference-based cell type annotation

If a reference dataset is provided, the cell type annotation can be transferred by the Seurat reference mapping method to facilitate result interpretation [32, 33]. We also developed a separate tool, scRef, to easily convert a previously analyzed dataset to reference data, which ensures the flexibility and reproducibility of cell type annotation. This reference-building step converts the scRNA-seq data into an R object, following the standardized format of Azimuth reference (https://github.com/satijalab/azimuth-references) [32].

### Differential expression analysis

The differentially expressed genes between different phenotypically labeled within a cell group defined by either cell types or cluster annotations can be calculated using the following methods: NEBULA [58], glmmTMB [59], and MAST [60]. If needed, scRNASequest also offers pseudo-bulk-based differential expression (DE) analysis using DESeq2 [61], limma [62], and edgeR [63]. However, as the benchmarking study reported previously, NEBULA outperforms other methods in general [64]. Thus, the pipeline uses NEBULA to perform DE analysis by default, and the parameters for running this step are defined in the configuration file (Table 2). A second input file listing the pairwise comparisons is also required. A configuration file with default parameters and an empty comparison file containing only the header line are automatically generated at the first call of the pipeline with the path to the data folder as an argument. The user has to populate the comparison file, otherwise no DE analysis will be performed. The output of DE analysis includes a table summary of gene name, fold change, *p*-value and adjusted *p*-value, and related figures such as volcano plots. To facilitate DE analysis on previously analyzed data, we also incorporated a standalone DE function called scDEG.

### Standardized output

The pipeline generates h5ad files to store the final results. This file type is compatible with both Python and R interfaces and can be easily visualized through the cellxgene VIP platform [43, 44]. Furthermore, if the data meets the quality requirements, the analyst can publish the analysis result to the CellDepot [45] to facilitate data exploration and sharing, as well as long-term data management.

User provides gene expression UMI count matrix files (h5 or MEX) from Cell Ranger and sample metadata to the semi-automated workflow, scRNASequest. It generates basic quality control (QC) reports and allows users to choose from popular data harmonization tools such as Harmony [35], LIGER [36], and Seurat [32, 33] to remove batch effects. Azimuth [32] reference-based cell label transfer is enabled as optional to perform cell type identification. Cluster- or cell-type-specific multi-sample multi-condition single cell level DE analysis is by default performed through NEBULA [58]. Finally, an h5ad file will be generated to be loaded into the cellxgene VIP [43, 44] framework or CellDepot [45] single-cell data management system for interactive visualization and analysis. The main script for the analysis is scAnalyzer. Five additional scripts are also included in the scRNASequest pipeline suite: scRMambient, scTool, scRef, sc2celldepot, and scDEG.

## Results

### Highlights of scRNASequest

The main program of the scRNASequest pipeline is scAnalyzer, which performs a variety of single-cell/single-nucleus data processing and analysis functions. Key strengths of this pipeline include:

- 1) Semi-automated with a minimal number of input files.
- 2) Fast and efficient data processing powered by Python.
- 3) Allowing immediate visualization of the QC report for users to fine-tune the filtering parameters.
- 4) Providing harmonization results in a single run: Seurat, Harmony, and LIGER, coupled with kBET and Silhouette metrics to evaluate the results.
- 5) Ensuring seamless connections with other tools such as cellxgene VIP and CellDepot.

### Performing QC and cell filtering on a sample dataset

**Fig. 2:**
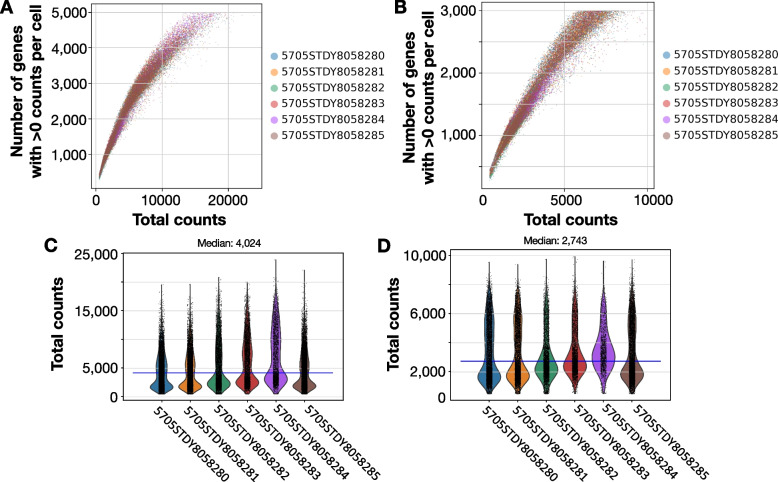
QC plots of the number of genes with greater than 0 counts per cell and total counts, before and after the filtering step. Scatter plot of total counts (X-axis, using the total_counts parameter from Scanpy QC metrics) and the number of genes (Y-axis, using the n_genes_by_counts parameter from Scanpy QC metrics) detected before (**A**) and after (**B**) filtering out low-quality cells and lowly expressed genes. Violin plots for each individual sample before (**C**) and after (**D**) filtering. The blue lines in **C** and **D** show the median counts of all data, with the exact number on top of the plots

We used a previously published dataset [65] to illustrate the QC step of scRNASequest. This mouse brain single-nucleus RNA-seq data contains six samples in total, three of them were from one mouse brain, and the other three were collected from another mouse. First, the pipeline generated the scatter plot of the number of genes detected versus the total counts, where n_genes_by_counts refers to the number of genes with at least one count in a cell) (Fig. 2A). It applied the default filtering criteria (min.cells = 3, min.features = 50, highCount.cutoff = 10,000, highGene.cutoff = 3,000) to generate the corresponding post-filtering plot (Fig. 2B). It also produced violin plots to provide an intuitive view of the pre- and post-filtering total count per cell distributions across samples (Fig. 2C, D). In addition, this QC step generated a variety of other plots, including the expression of the top 20 highly expressed genes, the percentage of mitochondrial reads, the percentage of reads mapped to various genomic regions (genome, gene, exonic regions, intronic regions, intergenic regions, antisense to gene regions, and transcriptome), and the percentage of Q30 bases in different read regions (barcode, RNA read, sample index and UMI). Finally, a summary was compiled with the number of cells and genes before and after each filtering step (Table 3). As expected, we observed decreasing cell counts and gene numbers due to the filtering steps, keeping only the cells of good quality. In this demo dataset, we eventually acquired 29,511 cells (in this case, nuclei) covering 25,115 genes for the downstream analysis.

### Delivering QC metrics through a Bookdown report

**Fig. 3:**
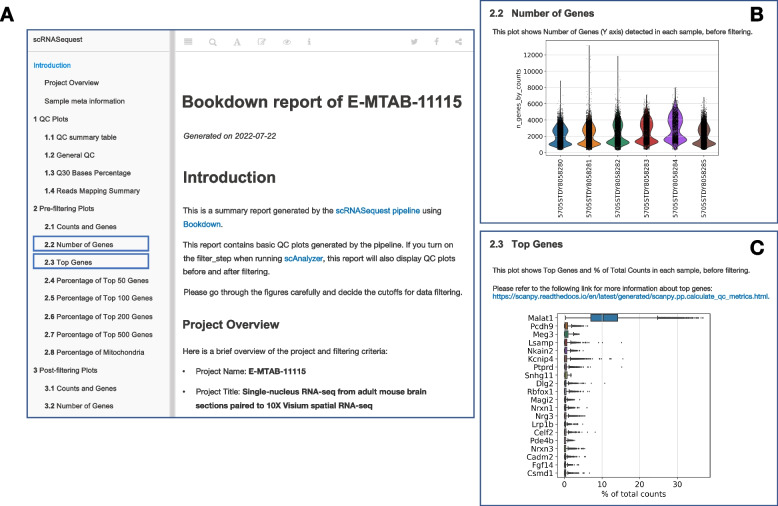
Bookdown report. **A** Bookdown document generated from the mouse brain snRNA-seq data, showing the preface. Each individual item on the left leads to related plots or tables. Clicking the items on the navigation bar will open corresponding pages. Two highlighted examples are in panels **B** and **C**. **B** An example of the ‘Number of Genes’ plot layout. **C** An example of the ‘Top Genes’ plot layout

Besides the h5ad and RDS files with the processed data, the pipeline generates a report to convey the complex concepts of the scRNA-seq analysis and provide immediate access to the results. This document is generated using the bookdown R package and includes the key tables and figures generated by the scRNASequest workflow. Bookdown originated from R Markdown and is dynamically generated, with code and figures embedded together in a book layout [42]. The bookdown document generated by our pipeline is an interactive webpage in HTML format, allowing the user to explore the results in a web browser without installing any specific software. It contains a high-level summary of the project, such as the data quality, mapping metrics, cell distribution, and top gene expression values before and after filtering. A snapshot of the bookdown report is shown in Fig. 3A. Further, it can be hosted on GitHub, as exemplified here (https://tinyurl.com/bdebvdz4), for broad sharing, especially in publications. In this report, the figures were organized into three different sections: 1) QC plots, 2) Pre-filtering plots, and 3) Post-filtering plots. It offers the flexibility to switch to and visualize different plots by selecting the items of interest from the left menu bar (Fig. 3B-C).

### Presenting the results to collaborators with the slide deck

Powered by reveal.js, an R markdown template is utilized by scRNASequest to generate an interactive slide deck with an emphasis on the graphical representation of QC and basic analysis plots. This makes the primary analysis presentation-ready for engaging biologists to discuss the initial results immediately after the pipeline run is finished. This slide deck can be opened using a web browser without the need for installing other software. It also allows the users to add notes to the slides or navigate to any other pages easily using the buttons on the corners. A full example of the slide deck can be found at the following link: https://tinyurl.com/bdepwy69.

### Data harmonization

**Fig. 4:**
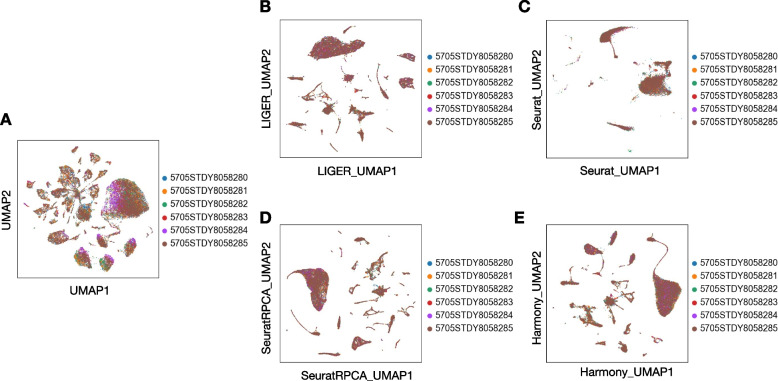
UMAP plots before and after four harmonization methods. **A** Original UMAP of six samples without harmonization. UMAPs of four harmonization methods: **B** LIGER, **C** Seurat, **D** Seurat RPCA, and **E** Harmony

Due to the high prevalence of batch effects in scRNA-seq datasets, data harmonization is critical when integrating multiple datasets and enables the identification of biological differences instead of technical ones. Through running scRNASequest, we can retrieve the results from different harmonization tools easily using the output h5ad file. To compare the outputs of these methods side-by-side, UMAP embedding plots were used to visualize the cells before and after harmonization (Fig. 4A-E) [66]. The pipeline also generated kBET and silhouette metrics (Fig. S1A-B) to quantify the performance of each harmonization strategy [55]. Lower kBET scores and higher silhouette coefficients are better. Based on the demo dataset, LIGER turned out to be the best tool to harmonize multiple data together (Fig. S1A-B).

### Cell type label transfer

**Fig. 5:**
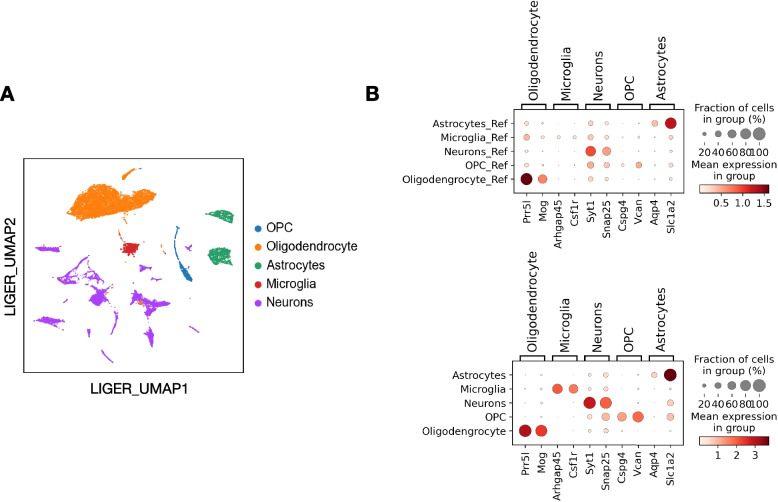
Reference-based cell type label transfer. **A** LIGER UMAP with five major cell types annotated. **B** Top marker genes for each cell type, in the reference dataset (upper panel) and query dataset (lower panel)

Automated cell type label transfer provides a fast and reproducible way to interpret the scRNA-seq results. We employed Seurat reference-based label transfer to annotate the cell types in our data. As a result, the algorithm identified major cell types in the mouse brain, including astrocytes, microglia, neurons, oligodendrocytes, and oligodendrocyte progenitor cells (OPCs). We used the UMAP after LIGER harmonization to present the label transfer results (Fig. 5A), with five major cell types separated clearly. We validated the correctness of the label transfer by identifying the most highly expressed genes in each cell type. We computed the top three genes within each cell type using the Scanpy rank_genes_groups function and presented the results in dotplots, comparing both reference dataset and query dataset (Fig. 5B). Many of these genes have been used as cell type marker genes in PanglaoDB [67]. For example, *Mog* is a marker gene for Oligodendrocytes, *Csf1r* has been used for microglia, *Cspg4* and *Vcan* are markers for OPC. Similarly, *Slc1a2* is a commonly used marker for astrocytes, while *Syt1* is a neuronal cell marker. Due to the heterogeneity of neurons, we did not expect to see any neuron subtype specific markers showing up as top marker genes in this analysis.

### Differential expression (DE) analysis

**Fig. 6:**
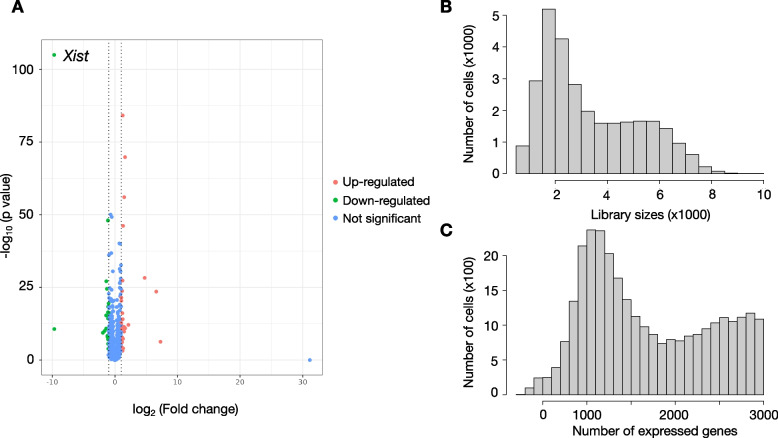
Differential expression analysis of male versus female in astrocytes. **A** Volcano plot of all genes with up- and down-regulated genes highlighted, respectively. Up-regulated genes were defined as genes with FDR < 0.05 and log_2 _FC > 1, and down-regulated genes were defined as genes with FDR < 0.05 and log_2 _FC < -1. **B** Histogram of total UMI counts. **C** Histogram of the number of expressed genes in each cell

Differential expression analysis is critical for identifying molecular differences underlying different conditions. Based on a recent benchmarking analysis, NEBULA has been identified as an ideal DE analysis method in general [64]. In scRNASequest, we incorporated NEBULA as the default method for the DE analysis step [58]. However, users can also choose from a number of DE methods we have implemented in the pipeline, including glmmTMB [59] and MAST [60]. In our demo dataset, we performed DE analysis on each cell type by comparing male versus female samples. *Xist*, the X-inactive specific transcript gene on the X chromosome, was identified as the most significantly decreased gene in males, which provides a proof-of-concept example of the DE analysis (Fig. 6A, Table S2) [68]. In addition to the DE gene table and the volcano plot, the pipeline also provides QC figures to display the total UMI counts and the number of expressed genes in the cells included in the comparison (Fig. 6B-C, Fig. S2-3).

### Reference building using scRef

Label transfer extracts the cell-type-specific gene expression signatures from previously analyzed datasets and applies the identified patterns to the new data. This step is critical for scRNA-seq data analysis, since cell-type labeling facilitates functional dissection and biological interpretation. However, the lack of proper references with matching cell type composition to the analyzed dataset can pose a challenge. Here, we provide scRef, a standalone functionality to process previously analyzed public datasets and embed their labeling information into a formatted reference file. This reference can then be used as input to the scRNASequest pipeline and guide the label transfer using pre-existing knowledge. scRef enables the user to bypass the laborious and limited cell-type annotation process of using only a small set of marker genes and enhances the efficiency and reproducibility of the analysis.

### Data visualization and exploration using Cellxgene VIP

**Fig. 7:**
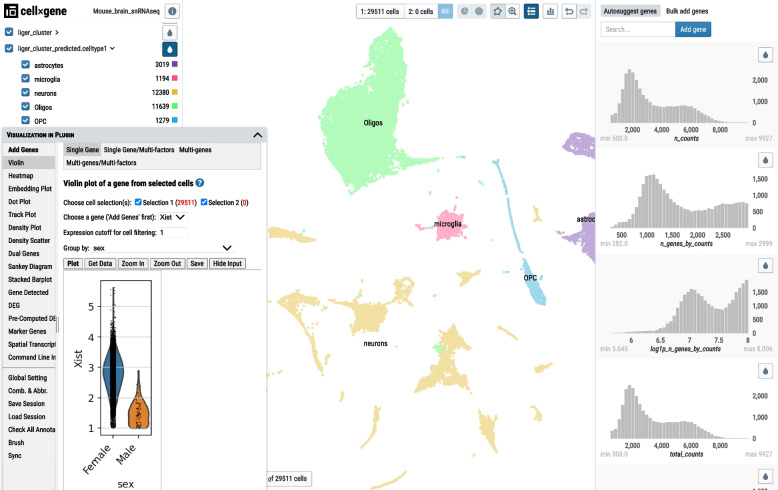
Cellxgene VIP interface. The ‘Visualization in Plug-in (VIP)’ window on the bottom left corner provides various plotting functions to navigate the full dataset

Cellxgene VIP is a plug-in tool to generate various figures for the processed dataset [43]. It was developed based on the cellxgene [44] single-cell visualization platform that displays categorical and numeric metadata information, as well as UMAP and PCA embedding plots. To facilitate smooth navigation of the dataset for scientists without programming backgrounds, scRNASequest generates an h5ad file that can be easily loaded into cellxgene VIP [43]. While cellxgene only provides a limited set of functions to explore the data, cellxgene VIP provides an expanded array of features: violin plots, volcano plots, dot plots, heatmaps, and complex figures in this all-in-one plug-in. As an example, we show that the *Xist* gene indeed has lower expression in the male compared to the female (Fig. 7). Detailed tutorial of the cellxgene VIP functionalities can be found on the tutorial page (https://interactivereport.github.io/cellxgene_VIP/tutorial/docs/).

### Integrating public datasets using sc2celldepot

We developed an API to load the scRNASequest result into a web-based application, CellDepot [45], designed to manage datasets from multiple projects and enable cross-dataset queries to create gene expression profiles in violin or dot plot formats. CellDepot also offers advanced search and filtering functions to locate datasets of interest quickly. To allow seamless export of scRNASequest outputs to CellDepot, the pipeline generates h5ad files containing all the necessary project and data information. However, it would be unnecessary and tedious work to run the entire pipeline on a previously analyzed public dataset (e.g., with cell type already labeled). Thus, we developed a functionality called sc2celldepot to easily convert public datasets into the required format and publish them into CellDepot.

## Discussion

With the nearly exponentially growing amount of scRNA-seq data generated, having a standardized and automated analysis pipeline is critical for efficiently processing and interpreting such data. To fulfill this need, we developed scRNASequest by incorporating multiple state-of-the-art tools into a scRNA-seq analysis pipeline with full integration between its component functionalities. Compared to other scRNA-seq analytic workflows, including scFlow [40], nf-core/scrnaseq [69], single-cell-rna-seq (https://github.com/snakemake-workflows/single-cell-rna-seq), scRNAseq_KNIME [70] and ASAP (https://github.com/DeplanckeLab/ASAP), scRNASequest offers several advantages (Table S1). First, scRNASequest uses clearly defined configuration files to set up analysis, allowing the users to fine-tune the parameters for specific steps. Also, its output results are compatible with multiple downstream tools and platforms, saving time for cross-platform data conversion. Moreover, we not only offer analysis pipelines but also provide cellxgene VIP and CellDepot as data visualization and management tools, making scRNASequest an ecosystem for scRNA-seq data analysis.

Interactive visualization has been a crucial component for interpreting scRNA-seq results, and several tools and platforms have been developed to meet this need, such as iSEE [71], scSVA [72], SCope (https://github.com/aertslab/Scope), etc. [73]. We performed a systematic comparison demonstrating the diverse features of cellxgene VIP and existing tools (Table S3). Overall, cellxgene VIP shows advantages in interactive data analysis and flexibility in web sharing.

Given the rapidly evolving nature of single-cell omics technologies, we are devoted to implementing new features of scRNASequest in the future. We plan to: 1) support single-cell multimodal omics data analysis such as scATAC-seq and spatial transcriptomics, 2) incorporate trajectory analysis using Monocle3 and RNA Velocity, 3) implement cell–cell communication inference tools, and 4) allow more quality control features such as doublet/multiplet removal.

## Conclusion

scRNASequest offers a one-stop-shop for analysts to process scRNA-seq data starting from UMI counts in either h5 or MEX format, perform harmonization of data from multiple samples, annotate cell types based on reference data, identify differentially expressed genes, and generate a suite of interactive reports in easy-to-access formats to enable biologists without advanced computational skills to explore the data interactively. With several user-friendly features such as the bookdown report, slide deck presentation, and cellxgene VIP exploration, users have the flexibility to analyze and share the results in multiple ways. The seamless integration with CellDepot makes data management and sharing possible for a collection of such datasets.

In summary, scRNASequest provides a user-friendly end-to-end pipeline for single-cell RNA-seq data analysis, visualization, and publishing to empower biologists to gain insights from high-volume sequencing data in digestible forms.

## Supplementary Information

**Additional file 1: Supplementary Table S1.** Detailed comparison of multiple single-cell RNA-seq data processing workflows.

**Additional file 2: Supplementary Table S2.** An example of NEBULA differential expression analysis results. FDR: False discovery rate. The NEBULA-HL method was used.

**Additional file 3: Supplementary Table S3.** Detailed comparison of multiple single-cell RNA-seq data visualization software.

**Additional file 4: Fig. S1.** Evaluation metrics for harmonization. (A) Batch correction performance evaluation by plots of kBET metrics and (B). Silhouette coefficients. For our demo dataset, LIGER was identified as the best batch correction method. **Fig. S2.** Percentage of top 50 features. **Fig. S3.** Number of cells with UMI > 0 for each gene (left) and the percentage of cells with UMI > 0 for each gene (right).

## Data Availability

Project name: scRNASequest. Project home page: https://github.com/interactivereport/scRNASequest Operating system(s): Linux system, MacOS. Software prerequisite(s): Conda or Docker. Programming language: Bash, Python, R License: MIT license. Any restrictions to use by non-academics: MIT license. Single-nucleus RNA-seq data: E-MTAB-11115 (ArrayExpression accession) and GSE185538 (GEO accession).

## Abbreviations

scRNA-seq: Single-cell RNA-seq
HPC: High-performance computing
NGS: Next-generation sequencing
QC: Quality control
DE: Differential expression
UMI: Unique molecular identifier
FDR: False discovery rate
PCA: Principal component analysis
RPCA: Reciprocal principal component analysis
UMAP: Uniform Manifold Approximation and Projection
kBET: K-nearest neighbor batch effect test
OPC: Oligodendrocyte progenitor cells
Cellxgene VIP: Cellxgene visualization in plug-in
