# Supplementary material for: scRNASequest: an ecosystem of scRNA-seq analysis, visualization, and publishing
Source: BMC Genomics. 2023 May 2;24:228. doi: 10.1186/s12864-023-09332-2 (PMC10155351; doi:10.1186/s12864-023-09332-2)
Supplement: Supplementary file 4 — Additional file 4: Fig. S1. Evaluation metrics for harmonization. (A) Batch correction performance evaluation by plots of kBET metrics and (B). Silhouette coefficients. For our demo dataset, LIGER was identified as the best batch correction method. Fig. S2. Percentage of top 50 features. Fig. S3. Number of cells with UMI > 0 for each gene (left) and the percentage of cells with UMI > 0 for each gene (right). [file 12864_2023_9332_MOESM4_ESM.pdf]

Fig. S1

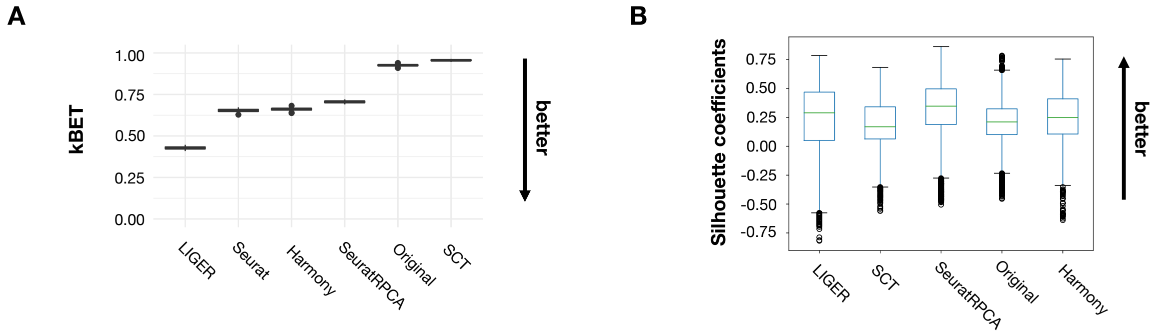

Fig. S1 Evaluation metrics for harmonization.
(A) Batch correction performance evaluation by plots of kBET metrics and (B) Silhouette coefficients. For our demo dataset, LIGER was identified as the best batch correction method.

Fig. S2

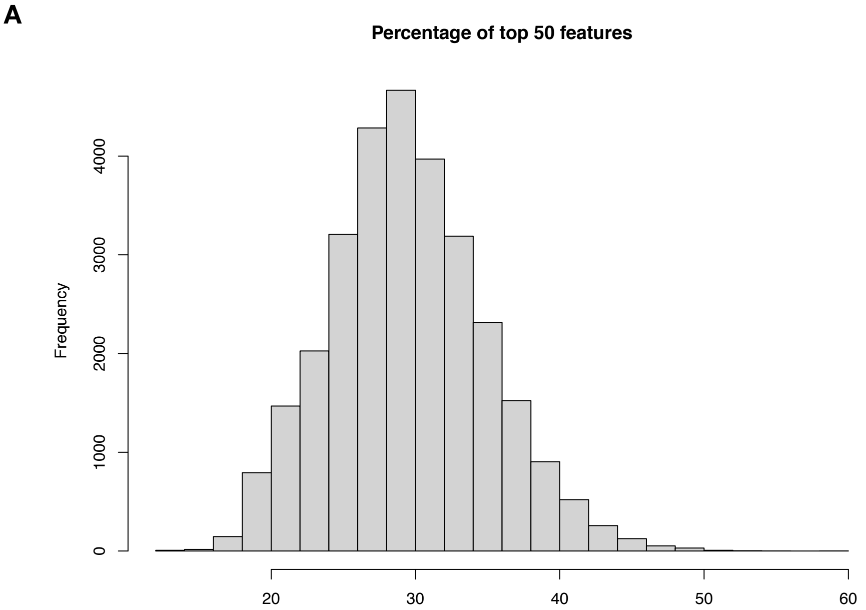

Fig. S2 Percentage of top 50 features.

Fig. S3

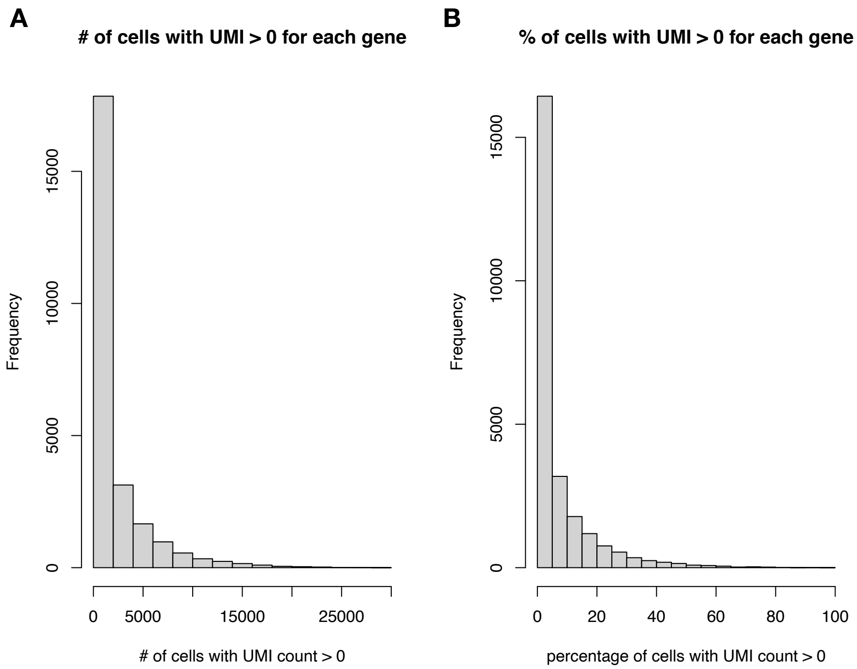

Fig. S3 Number of cells with UMI > 0 for each gene (left) and the percentage of cells with UMI > 0 for each gene (right).
